# Clinical efficacy of scaling and root planing with and without metronidazole on glycemic control: three-arm randomized controlled trial

**DOI:** 10.1186/s12903-021-01620-1

**Published:** 2021-05-12

**Authors:** Ambrina Qureshi, Syed Akhtar Hussain Bokhari, Zeba Haque, Akhtar Ali Baloch, Sidra Zaheer

**Affiliations:** 1 Department of Community and Preventive Dentistry, Dow University of Health Sciences, Ojha Campus, Karachi, Pakistan; 2 Department of Preventive Dental Sciences and Department of Postgraduate Studies and Scientific, College of Dentistry, King Faisal University Al-Ahsa, Al Hofuf, 31982 Kingdom of Saudi Arabia; 3 Department of Biochemistry, Dow International Medical College, Dow University of Health Sciences, Ojha Campus, Karachi, Pakistan; 4 National Institute of Diabetes & Endocrinology, Dow University of Health Sciences, Ojha Campus, Karachi, Pakistan; 5 Department of Research & Biostatistics, School of Public Health, Dow University of Health Sciences, Ojha Campus, Karachi, Pakistan

**Keywords:** Periodontitis, Diabetes, Clinical trial(s), Non-surgical periodontal therapy

## Abstract

**Background:**

Treating periodontitis through non-surgical periodontal therapy (NSPT) may improve glycemic control in type-2 Diabetes Mellitus (T2DM) patients. However, the evidence to maintain this improvement beyond four months is insufficient. Hence, this trial was conducted to assess clinical efficacy of NSPT on glycemic control in T2DM patients.

**Methods:**

This three-arm randomized controlled trial recruited 150 known T2DM participants (35–65 years), suffering from moderate to severe periodontitis, having HbA1c level ≥ 6.5% at baseline. Participants were followed up at 3 and 6 months. Intervention for test group-1 included scaling and root planing (SRP) with metronidazole (MET) and oral hygiene instructions (OHI). Test group-2 was intervened with SRP + OHI and control group with OHI only. Stata v. 14 was used to observe inter and intragroup mean changes in glycemic [glycated hemoglobin (HbA1c), fasting blood glucose (FBG)] and periodontal variables [bleeding on probing (BOP), periodontal pocket depth (PPD), clinical attachment loss (CAL)] using ANOVA and RMANOVA. Proportion of change in outcome variable (HbA1c) was assessed between treatment groups using chi-square test. Change was considered significant at *p*-value ≤ 0.05.

**Results:**

A significant reduction was observed in BOP, PPD, CAL, HbA1c and FBG over time [*p* < 0.05]. Significant reductions were observed in same variables in both test groups in comparison to control arm [*p* < 0.05]. No change between the two test groups was observed [*p* > 0.05].

**Conclusion:**

Scaling and root planing improves glycemic control of T2DM patients independently of the use of MET. Therefore, SRP after every 6 months may be suggested and included as a part of overall diabetes management for patients suffering from T2DM.

**Clinical trial registration** NCT 03,343,366 [Date of Registration: 17/11/2017]

## Introduction

Research on systemic sequel of periodontitis has remained an area of particular interest in dentistry. More than a decade ago meta-analysis of 57 peer-reviewed studies suggested that periodontitis may have a causal relationship with systemic diseases such as diabetes mellitus (DM) [1]. This was based on a hypothetical stance that inflammatory and microbial cells and their byproducts may prompt the acute production of inflammatory cytokines, interleukins and prostaglandins that impair insulin sensitivity or action over a period of time [2]. Cochrane presented a systematic review in 2015 and suggested that treating periodontitis may improve glycemic control of DM patients [3]. Their main outcome was a significant post-periodontal therapy reduction of HbA1c level in type-2 DM (T2DM) patients after 3 to 4 months. Nevertheless, the evidence was insufficient regarding the reduction in HbA1c level maintained beyond 4 months. Further research was recommended to evaluate adjunct drug therapies included as periodontal treatment, along with an extended follow up period and addition of third “no treatment” control group. They further added that the researches in this area were small in number and underpowered.

To carry out a research using prophylactic antibiotic as an adjunct therapy may not have been an appropriate choice of intervention in persons who are suffering from periodontitis having an otherwise normal general health condition. But in patients with co-morbidities the choice may be different. A pool of studies assessing scaling and root planing (SRP) + systemic antibiotic v/s SRP alone showed an inconsistent evidence towards improved glycemic control in patients suffering from uncontrolled T2DM [3]. However, individual studies suggested that systemic antibiotics may be prescribed while balancing against their side effects [4–8]. Recently, Souto et al. have systematically reviewed and found that amoxicillin (AMOX) + metronidazole (MET) have provided best clinical results in non-surgical periodontal therapy (NSPT) [9]. According to WHO it is recommended that broad-spectrum antibiotics or combo antibiotics must be avoided except in cases of severe infection that does not respond otherwise [10]. Metronidazole (MET), which is considered to have a narrow-spectrum has shown its clinical significance in the reduction of periodontal pocket depth (PPD) in patients suffering from periodontitis [11]. Another study, followed up to 12 months, showed significant benefit of using MET 400 mg × 3 for 10 days in case of patients having ≥ 5 mm PPD without any significant side effect [12]. As researchers have been observing the effects of MET, thus its use may be considered in diabetic patients for reduction in HbA1c level without any serious harm.

This trial was planned due to limited and inconsistent evidence about the effectiveness of MET in addition to SRP with regard to any improvement in T2DM. Moreover, the current research was undertaken to fill the research gap identified by the reviews which included (a) lack of large multi-centric trials consisting of participants with higher levels of uncontrolled glycemic values [3, 13–15], (b) imbalanced randomization and lack of allocation concealment[3] and (c) covariates and mediating factors involved in pathway between DM and periodontitis to be considered.

The objective was to assess the impact of SRP with and without MET on the glycemic control of T2DM patients at 3 and 6 months of intervention. The null hypothesis that there is no post intervention difference in the glycemic level (HbA1c and FBG) was considered to be rejected at *p*-value ≤ 0.05.

## Methods:

A parallel group, three-arm RCT was conducted at Dow University of Health Sciences Karachi which is one of the largest tertiary care hospital settings in Pakistan. The periodontal examiners were masked about the laboratory results and the laboratory personnel were masked about the examined periodontal results. Ethical approval was sought before the commencement of the trial by the Ethical Review Committee of the Institutional Review Board, Dow University of Health Sciences *[Ref. No.: IRB-900/DUHS/Approval/2017/146]*. The research protocol was registered with the Protocol Registration and Results System at ClinicalTrials.gov *[NCT 03343366]* on 17/11/2017[16] following the CONSORT guidelines [17]. The sample size estimation, screening, eligibility assessment, baseline laboratory and periodontal investigation are described in detail elsewhere [18]. Minimum sample size determined was n = 105 with 35 participants in each group with a ratio of 1:1:1 [15], however, the number was increased to 150 participants. Informed written consent was taken from each trial participant prior history taking, examination and trial procedure at baseline as well as on each follow-up. Individuals were included if they had ≥ 2 interproximal sites having ≥ 5 mm PPD or ≥ 4 mm of clinical attachment loss (CAL) [19]. The periodontal examination was performed and recorded by two trained and calibrated examiners. Inter-examiner agreement for PPD and CAL were calculated at preliminary stage of the study and was found as 82.73% with Cohen’s Kappa value = 0.456 [*p* < 0.001] for PPD, and for CAL was 88.18% with Cohen’s Kappa value = 0.649 [*p* < 0.001].

### Enrollment and randomized allocation

After confirming age of the participants (35–65 years), with at least 16 natural teeth on examination, having moderate to severe periodontitis, HbA1c level ≥ 6.5% and < 14% at baseline with already diagnosed T2DM since ≥ 1 years were enrolled in the study. Patients under either or both kind of diabetes management (insulin and/ or oral glycemic therapy) were also considered as included. Initially it was planned to include participants with HbA1c level ≥ 6.5% and < 10% [16]. However, due to majority of patients seeking diabetes care at the center were with poorly controlled diabetes with higher levels of HbA1c willing to get enrolled in trial we had to change the maximum cut-off level of HbA1c to < 14%. All participants were requested to read, understand and sign a written consent to participate in the trial. Eligible participants were randomly allocated to either of the two NSPT test groups including SRP + MET or SRP and control group through simple randomization scheme using computer generated random number table. Random allocation was performed by an independent allocator using Sequentially Numbered and Opaque Sealed Envelopes (SNOSE) containing detailed instructions for each intervention that were opened only by the chair side dental assistant. These envelops were kept confidential and sent back to the allocator by the dental assistant which were disclosed at the time of statistical analysis to check the type of intervention performed.

### Intervention

Interventions were provided by trained periodontal therapists who were masked and unaware of the examined periodontal and glycemic findings. Similarly, the periodontal examiners and biochemist were unaware of the type of intervention performed by the periodontal therapists. The NSPT was performed within 10 days after inclusion in study. All participants were given general dietary and healthy lifestyle information at each follow-up. Demonstrations about OHI were provided by trained oral health educators. All patients were instructed to brush their teeth using soft toothbrush and fluoridated toothpaste twice daily (morning after breakfast and night before sleeping) using modified bass technique. This was reinforced on each follow-up visit.

Participants of test group-1 received SRP through a combination of ultrasonic scaling (average 60 min on medium intensity full mouth in single sitting) and hand instrumentation (using sharpened and sterilized curettes) to smoothen irregular areas of root surface until the surfaces were smooth[20] followed by MET 400 mg × 3 for 10 days[12] along with warm salt water rinses for 3–5 days[21] and OHI. Participants in test group-2 received the same intervention as test group-1 except MET. The control-group received only OHI and was provided periodontal therapy at end of trial.

### Statistical analysis

Data was entered using statistical software Stata v. 11.0. After descriptive statistics, inferential analysis was performed using RMANOVA and ANOVA to assess within group and between groups mean differences. Mean differences were observed within each group from baseline to 1-month and 3-month follow-up for periodontal (BOP, PPD and CAL) and glycemic variable (FBG). Whereas, mean change in HbA1c which was the only outcome variable was assessed from baseline to 3-months and 6-months. Proportion of change in outcome variable (HbA1c) was assessed between treatment groups using chi-square test. Change was considered significant at *p*-value ≤ 0.05. Per protocol (PP) analysis was performed on data of only those participants who showed compliance with study protocol. Intent-to-treat (ITT) analysis was applied to assess any bias in the results due to attrition. Significant *p*-value was considered at 5% [*p* ≤ 0.05].

## Results

**Fig. 1:**
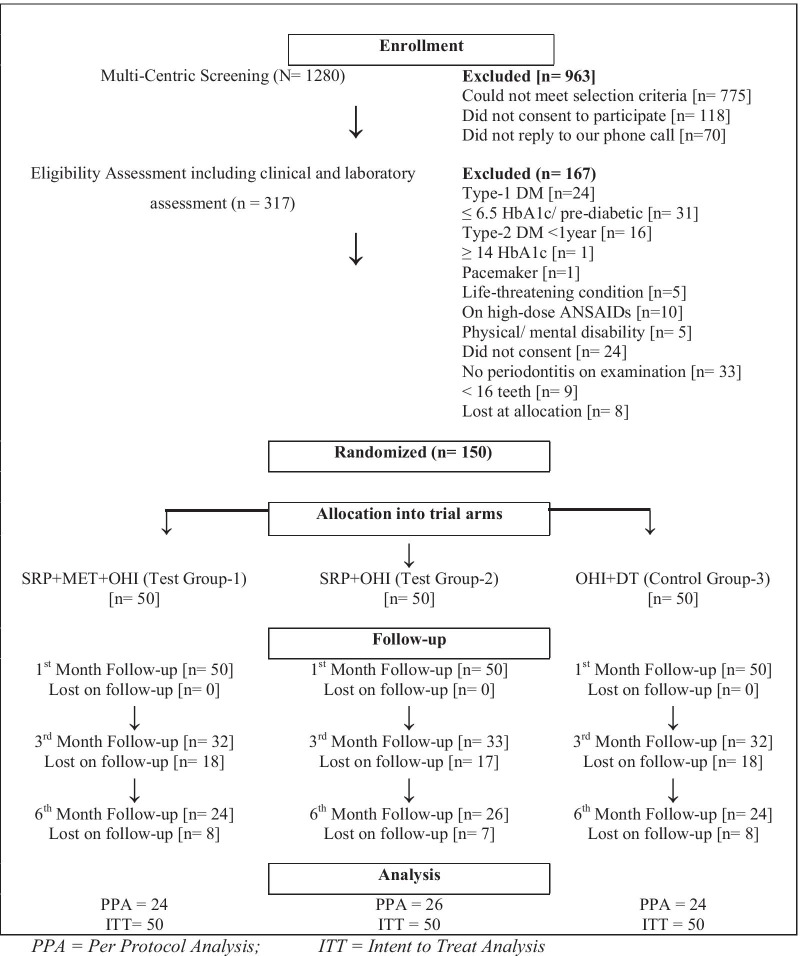
Study flow chart

Out of all screened individuals [N = 1280] at multi-centric camps, 963 were excluded. A total of 387 individuals were referred to the base camp for further eligibility assessment, 317 turned up to the base camp. Out of them, 167 individuals were further excluded (Fig. 1) and 150 participants were randomly allocated into study arms. On the 1^st^ follow-up visit by approximately 30 days [mean = 31.73 ± 4.55 days], 100% response was achieved. Out of 150 participants, 97 [64.66%] participants reported on 3-month follow-up. Further 23 participants were lost on 6^th^ month follow-up leaving behind total 74 participants with n = 24, n = 26 and n = 24 in the two test and control arms respectively.

Baseline characteristics of the 150 recruited participants allocated in three arms are presented in Table 1. There was no difference found in baseline variables among the three groups after allocation [*p* > 0.05].

**Table 1:** Baseline differences at the time of allocation in three-arms (n = 50 in each arm)

Variables	SRP + MET + OHI	SRP + OHI	OHI + DT	*p*-value
*Gender*				
Females Male	20 30	23 27	25 25	0.600
*BMI status*				
Underweight Normal Overweight Obese	2 15 25 8	0 15 28 7	3 14 27 6	0.770
*Smoking*				
No Yes	47 2	46 2	43 4	0.559
*Diabetic management*				
Hypoglycemic Insulin Both	37 4 9	31 9 10	38 6 6	0.427
*Co-morbidity*				
None Hypertension Others	29 13 7	25 10 14	26 10 13	0.643
*Regular exercise*				
No Yes	24 26	21 29	21 29	0.782
*Healthy diet*				
No Yes	10 40	15 35	13 37	0.512
*HbA1c (cut-off 9%)*				
< 9% ≥ 9%	25 25	29 21	29 21	0.649
Mean age (years)	52.72 ± 8.00	51.24 ± 8.27	52.82 ± 6.38	0.509
Education (years)	11.18 ± 4.97	10.69 ± 4.62	9.87 ± 5.52	0.540
Mean BMI (m/kg^2^)	26.66 ± 5.23	26.47 ± 3.61	25.72 ± 4.21	0.533
Total teeth	24.84 ± 3.15	24.88 ± 3.02	25.12 ± 3.27	0.891
Teeth indicated for extraction	0.97 ± 1.97	0.54 ± 1.11	1 ± 1.70	0.286
HbA1c (%)	9.11 ± 1.52	9.09 ± 1.75	8.88 ± 1.65	0.727
BOP (%)	26.03 ± 10.67	26.07 ± 9.57	26.25 ± 9.81	0.993
PPD (mm)	3.40 ± 0.88	3.56 ± 0.89	3.47 ± 0.86	0.644
CAL (mm)	3.94 ± 0.96	3.87 ± 1.04	3.80 ± 1.11	0.791

SRP, scaling root planning; MET, metronidazole; DT, delayed treatment; OHI, oral hygiene instructions; BOP, bleeding on probing; PPD, periodontal pocket depth; CAL, clinical attachment loss; mm, millimeter

Table 2 presents intra-group mean differences in the glycemic and periodontal variables from baseline (0) to subsequent post-intervention follow-up period of participants within each trial arm. Significant mean reductions were observed in all glycemic and periodontal variables in both test arms. Whereas in control arm, significant increase in mean HbA1c levels were observed at 3 and 6 months by 0.69% and 1.31% respectively [*p* < 0.05]. The mean PPD also significantly increased [*p* = 0.025] in control group, whereas BOP% and mean CAL remained unchanged [*p* > 0.05].

**Table 2:** Post intervention within group mean differences over time in glycemic and periodontal variables

Variables	SRP + MET + OHI (n = 24)	∆	*p*-value*	SRP + OHI (n = 26)	∆	*p*-value*	DT + OHI (n = 24)	∆	*p*-value*
HbA1c (0 m)	9.05 ± 1.70	–	** < 0.001**	9.05 ± 1.83	–	** < 0.001**	8.34 ± 1.26	–	**0.001**
HbA1c (3 m)	7.8 ± 1.25	− 1.25		8.05 ± 1.29	− 1.0		9.03 ± 1.23	0.69	
HbA1c (6 m)	7.47 ± 1.19	− 1.58		7.81 ± 1.43	− 1.24		9.65 ± 1.85	1.31	
FBG (0 m)	187.5 ± 56.69	–	** < 0.001**	180.80 ± 52.18	–	**0.006**	172.66 ± 54.34	–	0.425
FBG (1 m)	177.29 ± 54.70	− 10.21		167.46 ± 53.61	− 13.34		179.41 ± 49.80	6.75	
FBG (3 m)	147.71 ± 36.94	− 39.79		151.56 ± 35.80	− 29.24		181.04 ± 55.51	8.38	
FBG (6 m)	137.47 ± 44.96	− 50.03		147.79 ± 37.75	− 33.01		189.30 ± 51.81	16.64	
Mean BOP% (0 m)	25.79 ± 10.09	–	** < 0.001**	25.25 ± 8.63	–	** < 0.001**	22.08 ± 8.02	–	0.661
Mean BOP% (1 m)	13.65 ± 9.40	− 12.14		14.54 ± 6.01	− 10.71		21.81 ± 13.49	− 0.27	
Mean BOP% (3 m)	12.88 ± 7.86	− 12.91		11.66 ± 4.95	− 13.59		23.61 ± 12.82	1.53	
Mean PPD (0 m)	3.64 ± 0.88	–	** < 0.001**	3.44 ± 0.95	–	** < 0.001**	3.04 ± 0.70	–	**0.025**
Mean PPD (1 m)	2.45 ± 0.41	− 1.19		2.69 ± 0.68	− 0.75		3.17 ± 0.79	0.13	
Mean PPD (3 m)	2.29 ± 0.50	− 1.35		2.68 ± 0.82	− 0.76		3.43 ± 0.95	0.39	
Mean CAL (0 m)	4.20 ± 0.77	–	** < 0.001**	3.74 ± 0.94	–	** < 0.001**	3.55 ± 1.11	–	0.940
Mean CAL (1 m)	3.04 ± 0.57	− 1.16		2.97 ± 0.83	− 0.77		3.55 ± 0.94	0.00	
Mean CAL (3 m)	2.96 ± 0.70	− 1.24		2.54 ± 0.76	− 1.2		3.60 ± 0.91	0.05	

SRP, scaling root planning; MET, metronidazole; DT, delayed treatment; OHI, oral hygiene instructions

0 m = baseline, 1 m = 1-month, 3 m = 3-months, 6 m = 6-months

∆ Intra-group mean differences between baseline and each follow-up reading

*All Significant *p*-value < 0.05 measured by RMANOVA have been made bold

Table 3 presents post intervention between group analysis with respect to periodontal (BOP, PPD, CAL) and glycemic variables (HbA1c, FBG). Post therapy changes in periodontal variables were observed at 1 and 3 months. Post-hoc results showed that there was a significant reduction observed in all periodontal variables in test arms with respect to control arm [*p* < 0.05]. However, there was no significant difference change found in periodontal variables between two test arms [*p* > 0.05]. Post therapy changes in glycemic levels were observed at 3 and 6 months. Post-hoc results showed that there was a significant reduction in both glycemic variables in test arms with respect to control arm [*p* < 0.05]. However, there was no significant difference between test arm-2 (SRP + OHI) and control arm [*p* > 0.05] with respect to FBG level. Also, no significant difference between two test arms [*p* > 0.05] was found for glycemic variables.

**Table 3:** Post intervention changes in periodontal and glycemic variables at different follow-up visits

Variables v/s intervention	On 1-month follow-up			On 3-months follow-up		
Periodontal	Mean difference ∆	*p*-value*	*p*-value**	Mean difference ∆	*p*-value*	*p*-value**
*Mean BOP %*						
**∆** SRP + MET + OHI − DT + OHI	− 8.16	**0.010**	**0.018**	− 10.72	** < 0.001**	**0.001**
**∆** SRP + OHI − DT + OHI	− 7.27		**0.037**	− 11.94		** < 0.001**
**∆** SRP + MET + OHI − SRP + OHI	− 0.89		1.000	1.22		1.000
*Mean PPD* (mm)						
**∆** SRP + MET + OHI − DT + OHI	− 0.72	** < 0.001**	**0.001**	− 1.13	** < 0.001**	** < 0.001**
**∆** SRP + OHI − DT + OHI	− 0.48		**0.031**	− 0.74		**0.008**
**∆** SRP + MET + OHI − SRP + OHI	− 0.24		0.593	− 0.39		0.357
*Mean CAL* (mm)						
**∆** SRP + MET + OHI − DT + OHI	− 0.50	**0.028**	0.098	− 0.63	** < 0.001**	**0.032**
**∆** SRP + OHI − DT + OHI	− 0.57		**0.040**	− 1.05		** < 0.001**
**∆** SRP + MET + OHI − SRP + OHI	0.07		1.000	0.41		0.309
*Glycemic*	*On 3-months follow-up*			*On 6-months follow-up*		
*HbA1c (%)*						
**∆** SRP + MET + OHI − DT + OHI	− 1.22	** < 0.002**	**0.004**	− 2.18	** < 0.001**	** < 0.001**
**∆** SRP + OHI − DT + OHI	− 0.98		**0.023**	− 1.84		** < 0.001**
**∆** SRP + MET + OHI − SRP + OHI	− 0.24		1.000	− 0.34		1.000
*FBG* (mg/dL)						
**∆** SRP + MET + OHI − DT + OHI	− 33.32	** < 0.023**	**0.042**	− 51.82	** < 0.001**	**0.001**
**∆** SRP + OHI − DT + OHI	− 29.48		0.068	− 41.51		**0.007**
**∆** SRP + MET + OHI − SRP + OHI	− 3.84		1.000	− 10.31		1.000

SRP, scaling root planning; MET, metronidazole; DT, delayed treatment; OHI, oral hygiene instructions

∆Between groups mean difference

*All Significant difference between three groups [*p* < 0.05] measured by One-way ANOVA have been made bold

**All Significant difference at *p* < 0.05 calculated through post-hoc bonferroni test have been made bold

Table 4 shows PPA and ITT analyses pertinent to the proportion of participants whose HbA1c level was reduced to < 7% by 3 and 6 months of intervention in three trial arms. Chi-2 test results showed significant difference between participants with < 7% and ≥ 7% HbA1c levels at 3 months but the difference was not significant at 6 months. At 3 months follow-up maximum number of participants with < 7% HbA1c level were observed in test group-1 (SRP + MET + OHI) and none in control group. On the other hand, intervention with SRP + OHI showed maximum number of participants whose HbA1c levels were reduced to < 7% by 6 months but this result was not significant [*p* > 0.05]. The ITT analyzed result was not different from PPA result. None of the participants reported any side effects during the trial period.

**Table 4:** Proportion of change in outcome variable (HbA1c level at cut-off = 7%) after 3 months and after 6 months of intervention

Intervention groups	At 3rd month follow-up		*p*-value (chi2)*	At 6th month follow-up		*p*-value (chi2)*
	HbA1c < 7%	HbA1c ≥ 7%		HbA1c < 7%	HbA1c ≥ 7%	
*Per-Protocol (PP) analysis*						
DT + OHI	0 (0)	24 (100)	**0.020 (7.78)**	2 (8.33)	22 (91.67)	0.061 (5.60)
SRP + OHI	5 (19.23)	21 (80.77)		9 (34.62)	17 (65.38)	
SRP + MET + OHI	7 (29.17)	17 (70.83)		8 (33.33)	16 (66.67)	
*Intention-To-Treat (ITT) analysis*						
DT + OHI	1 (3.13)	31 (96.88)	**0.045 (6.22)**	2 (8.33)	22 (91.67)	0.061 (5.60)
SRP + OHI	5 (15.15)	28 (84.85)		9 (34.62)	17 (65.38)	
SRP + MET + OHI	8 (25.0)	24 (75.0)		8 (33.33)	16 (66.67)	

*All Significant *p*-value < 0.05 calculated by chi-2 test have been made bold

## Discussion

The surface area of inflamed periodontal tissues is almost the size of a palm of the human hand [22]. Hypothetically, if that much surface is partially or entirely infected with gross suppuration and inflammation, it would not be surprising to have a systemic consequence [23]. The current trial results are justifiable to draw a conclusion that managing periodontal disease through NSPT is efficacious in reducing not only periodontitis but also controlling the glycemic level in T2DM patients. It suggests that while treating periodontitis with any suitable therapy, the focus may be directed towards the disruption of sub-gingival calculus and plaque biofilms so that its clinical efficacy may be achieved [24]. Researchers have suggested that 2–3 mm changes are considered necessary to confidently determine that the change has occurred [25]. However, our results showed less than 2 mm change in PPD and CAL both with reference to the baseline measures within intervention arms as well in the control arm over a period of 1 and 3 months. Participant in control arm experienced hardly a millimeter change in PPD and CAL which could be the reason that no significant metabolic change was observed in this group. This justification is same as presented by Jones et al. in their study, where no impact on diabetes level was evident in their study groups over time due to delayed post therapy periodontal healing [26]. Comparing the existing periodontal results with that of another recent trial (PARODIA 1 Study) conducted among Sub-Saharan African population it was observed that they found 1.1 mm reduction in PPDand 1.3 mm reduction in CALafter 3 months of NSPT [27]. Whereas, over a period of 3 months our study found 0.76 mm and 1.35 mm significant reduction in mean PPDin both test trial arms respectively relative to that measured at baseline [Table 2]. Moreover, we found an almost 1.2 mm significant reduction in CALin both SRP + OHI and SRP + MET + OHI trial participants with reference to their baseline mean CAL. Reduction in CAL was also comparable to the same study where chlorhexidine (CHX) mouthwash was used as an adjunct to the intervention [27]. We did not use CHXmouthwash but we used warm salt water rinses in our study. As there was no differences in PPDand CALreduction between test group-1 (SRP + MET + OHI) and test group-2 (SRP + OHI), therefore it is suggested that there may be no additional requirement of MET as an adjunct antibiotic in the management of periodontitis. Miranda et al. on the other hand, found augmented benefit of MET + AMOX for periodontitits and other SRP related clinical and microbiological outcomes [28]. This may be because Miranda et al. used MET in combination with AMOX and not alone. However, reviews still suggest no additional requirement of AAT in the management of periodontitis [3]. Whatsoever, we suggest that alone SRPalong with OHI and warm salt water rinses instead of CHX mouthwash may be sufficient to reduce pocket depth and attachment loss to a reasonable extent over the period of 3 months. This effect may be due to sufficient plaque control ability of salt water rinses [21]. It is also important to highlight that in total there were only eight participants out of the total who were reportedly smokers. As there was no significant difference between the numbers of smokers in the three groups it may be suggested that effect of smoking on periodontitis could not be assessed in this study.

Our trial results showed that SRP with and without MET are significantly efficacious in reducing HbA1c levels as compared to OHI alone. Reduction in HbA1c level of > 1% over the period of 6 months was also in compliance with another study where T2DM patients were intervened with full mouth teeth extraction [29]. In this study a reduction of 1.23% and 1.37% in HbA1c level was observed after 3 and 6 months of intervention respectively. Therefore, it may be suggested that SRP with or without MET may be equally effective as is the full-mouth hopeless teeth extraction in improving glycemic control. However, this is not true with all clinical trials, which assessed the impact of periodontal therapy on glycemic control. Diabetes and Periodontal Therapy Trial (DPTT) conducted on a larger sample of over 500 patients failed to demonstrate any benefit of periodontal treatment on glycemic control neither at 3 months nor at 6 months [30]. However, this study has been criticized of recruiting patients with moderately good level of glycemic control and thus having limited potential for post PT glycemic improvement. So far, DIAPERIO trial that assessed the local and systemic effects of AMOX and SRP also recruited almost similar number of participants as that of ours [4]. However, they did not find any effective change in periodontal and glycemic measures over the period of 3-months. Similarly, they also did not observe any significant reduction in HbA1c in test arm after 3 months of therapy. The reason could be that majority of the participants belonged to T1DM and very few with T2DM. Moreover, the trial researchers admit that the study participants did not belong to true uncontrolled diabetic group and were already those who were enrolled in diabetes education program and belonged to high socio-economic status. The use of AAT is found to have a beneficial effect on the clinical and bacterial outcomes of SRP [28]. However, the evidence is found inconsistent in scientific pool of literature that addition of AAT has any additional effect SRP outcomes [3].

It is pertinent to mention here, that the present study participants were only T2DM patients and were found with a wider range of HbA1c levels between 6.5 and 14%. More than half of the recruited participants having poorly controlled HbA1c levels (> 8%) [31]. Recruiting patients with higher level of baseline HbA1c may be considered as strength of the study as this not only benefitted the participants to regularly get their glycemic status monitored during the trial period but also gave the investigators an opportunity to evaluate the effects through a range of hyperglycemic level.

Almost all reviews [3, 32, 33] have concluded that the trials on this topic were conducted on smaller samples which might have prevented the results to be extrapolated on population. As per our calculations, the minimum sample size required to observe a reduction of 0.6% in HbA1c with 90% power was 27 in each group. We raised our initial target sample to n = 50 in each arm by adding on 40% attrition as a number of barriers are linked with clinical trials conducted in developing countries [34]. This was the maximum attrition rate we could keep for the end-of-study endpoint. The idea was to enroll a higher number of patients such that an adequate sample size is achieved for the primary outcome measure which was HbA1c. Although the increased enrollment of participants resulted in an increased cost and delay in determining the study outcome, yet our focus was not to compromise the statistical power of this study that is usually expected in resource-limited settings [35]. Still large number of drop-outs at each follow-up may be considered as a major weakness of our study. Limitation of this study was that the investigators did not systematically keep a record of reasons for lost to follow-up of trial participants at 3 and 6 months. But our general observation was that the participants were unable to make up for follow up visits in fasting state which was required for their biochemical testing. Moreover, due to COVID-19 lockdown three of our trial patients missed out their last follow-up visit. The study could have been followed-up for more than 6 months but due to limitations linked with attrition subsequently leading to smaller sample size we could not carry on the follow-up further.

This study also took into account the baseline potential differences by randomly allocating the study sample into test and control groups. This was done to eliminate the selection bias, generate balanced groups with respect to confounding or prognostic variables and form a base for an assumption of a statistical test of equality of treatment. The baseline data with no statistical differences in the three arms may be considered as strength of this study. Moreover, while calculating proportion size of trial participants, it was found that around 30% of our participants in test group-1 and 20% in test group-2 significantly got benefitted by reduction of HbA1c level by < 7% within 3 months. Although this proportion was raised to more than 30% in both test groups by the end of 6 months but that was not significant. We found no difference between the results analyzed by PP and ITT which means there was no attrition bias in our study.

## Conclusion

The findings have revealed that SRP with or without MET is significantly efficacious in not only reducing periodontitis but also for glycemic control in T2DM patients suffering from higher levels of HbA1c at baseline. Therefore, SRP + OHI without added load of MET at 6 months may be suggested and included as a part of overall diabetes management. The trial results may facilitate in formulating an oral health integrated policy for the management of diabetic patients. However, further studies may be conducted to determine the effectiveness of NSPT with 12 months of follow-up.

## Data Availability

The datasets generated and analyzed during the current study are not publicly available as it is part of PhD research and may be available from the corresponding author on reasonable request only after thesis public defense.
